# Supplementary material for: Structures of Echovirus 30 in complex with its receptors inform a rational prediction for enterovirus receptor usage
Source: Nat Commun. 2020 Sep 4;11:4421. doi: 10.1038/s41467-020-18251-9 (PMC7474057; doi:10.1038/s41467-020-18251-9)
Supplement: Supplementary file 1 — Supplementary Information [file 41467_2020_18251_MOESM1_ESM.pdf]

Supplementary Information

Structures of Echovirus 30 in complex with its receptors inform a rational prediction for enterovirus receptor usage

Wang et al.

This file contains Supplementary Figures 1-11 and Supplementary Tables 1-3.

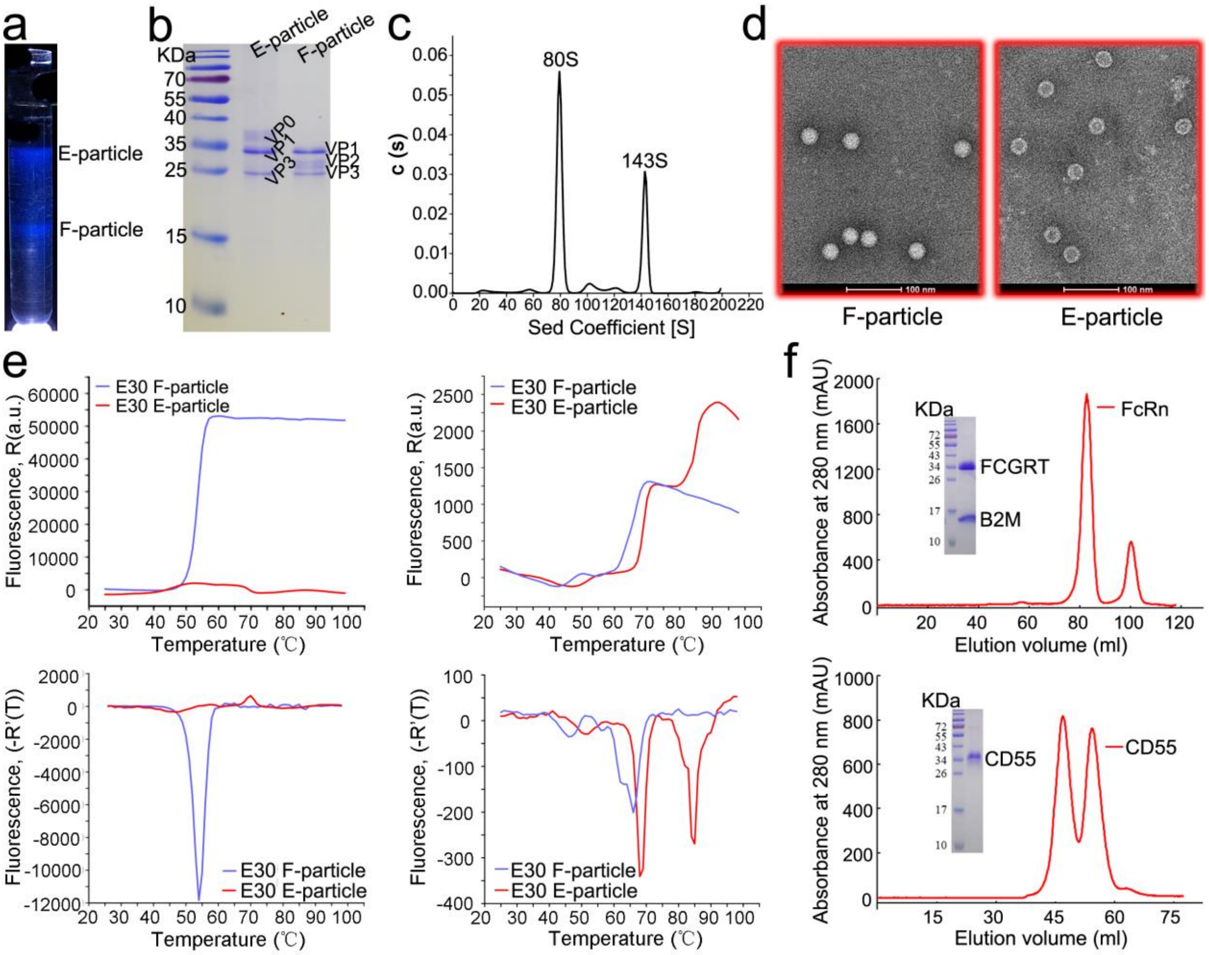

Supplementary Figure 1

Purification and characterization of E30. (a) Sucrose density gradient ultracentrifugation (from 15% to 45%) for the purification of E30, as described in Methods. Two marked bands, i.e., the top with an absorbance ratio of 1.7 mainly containing the F-particles, and the bottom with an absorbance ratio of 0.7, containing the E-particles could be identified. **(b)** SDS-PAGE analysis for viral protein composition. The estimated molecular weights of VP0, VP1, VP2, VP3 of E30 are 36 kDa, 32 kDa, 29 kDa, 26 kDa, respectively. **(c)** Analytical ultracentrifugation. The purified virus samples produced two major peaks with sedimentation coefficients of 80S and 143S corresponding to E30 F-particle and E-particle, respectively. **(d)** Negative stain images of E30 F-particles (left) and E-particles (right). **(e)** Thermofluor analysis of E30 F-particle and E-particle. We characterized the stability of E30 F-particle and E-particle using different dyes - SYTO9 (to detect viral exposed RNA; left) and SYPROred (to detect viral exposed hydrophobic residues; right). The derived raw fluorescence curves (upper) and their first derivatives (lower) indicate there is no RNA in E-particle, whereas the viral RNA in F-particle starts to release and quickly goes out completely within 15 degrees, with the exposure of some hydrophobic residues at ~ 45℃. And it turns out E-particle is

much more stable than F-particle as the former dissociates at 85℃, which is ~ 20 degrees higher than what E-particle needs. **(f)** The SDS-PAGE and gel filtration of the uncoating receptor FcRn (upper) and attachment receptor CD55 (lower).

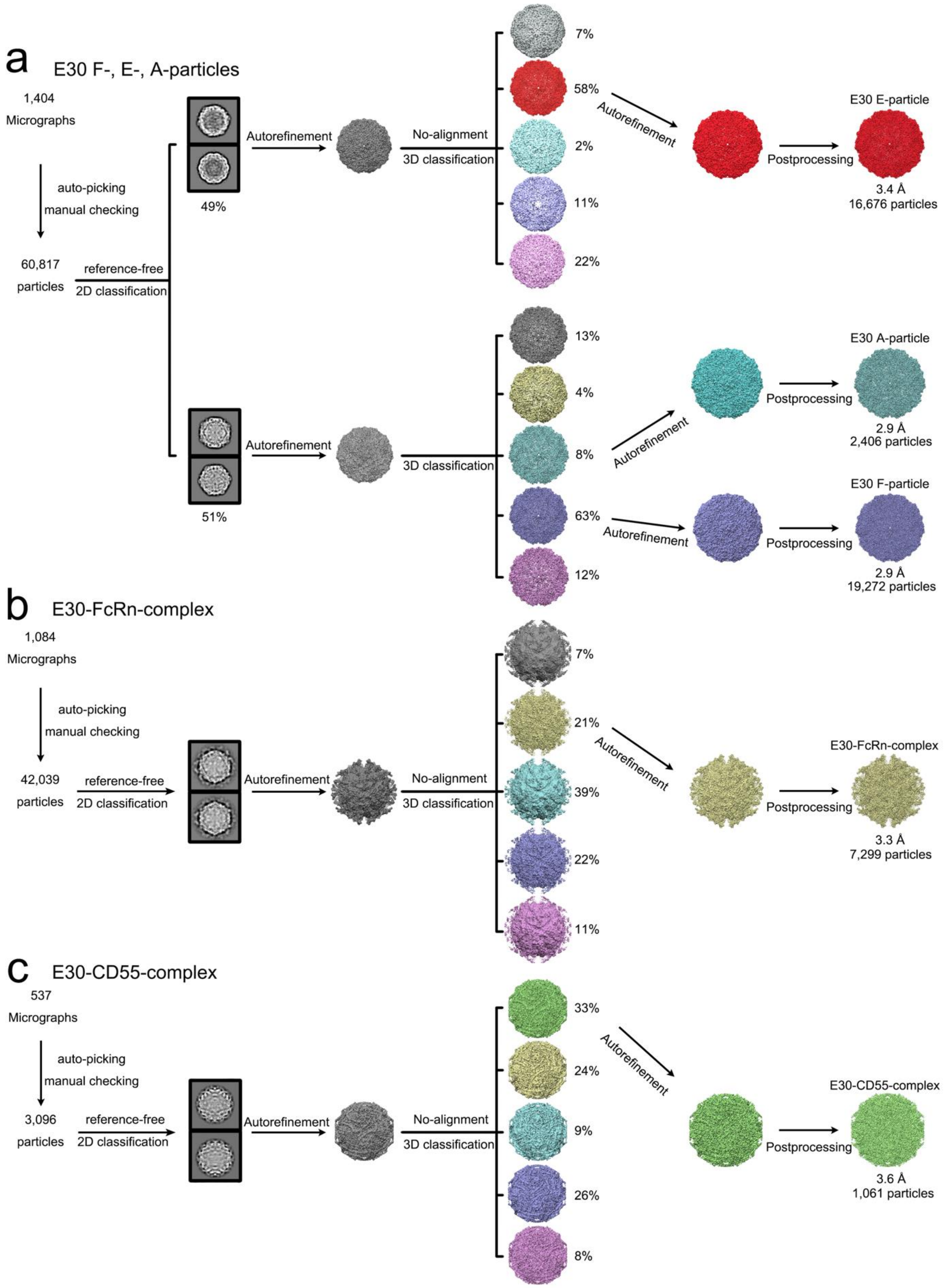
a
E30 F-, E-, A-particles
1,404
Micrographs
auto-picking
manual checking
60,817
particles
reference-free
2D classification
49%
Autorefinement
No-alignment
3D classification
7%
58%
2%
11%
22%
Autorefinement
E30 E-particle
Postprocessing
3.4 Å
16,676 particles
51%
Autorefinement
3D classification
13%
4%
8%
63%
12%
Autorefinement
E30 A-particle
Postprocessing
2.9 Å
2,406 particles
Autorefinement
E30 F-particle
Postprocessing
2.9 Å
19,272 particles
b
E30-FcRn-complex
1,084
Micrographs
auto-picking
manual checking
42,039
particles
reference-free
2D classification
Autorefinement
No-alignment
3D classification
7%
21%
39%
22%
11%
Autorefinement
E30-FcRn-complex
Postprocessing
3.3 Å
7,299 particles
c
E30-CD55-complex
537
Micrographs
auto-picking
manual checking
3,096
particles
reference-free
2D classification
Autorefinement
No-alignment
3D classification
33%
24%
9%
26%
8%
Autorefinement
E30-CD55-complex
Postprocessing
3.6 Å
1,061 particles

Supplementary Figure 2.

Flow-chart for cryo-EM data processing. (a), (b) and (c) show the data processing procedures for E30 particles, E30 F particles in complex with FcRn and E30 F particles in complex with CD55, respectively. Details can be found in the Methods section.

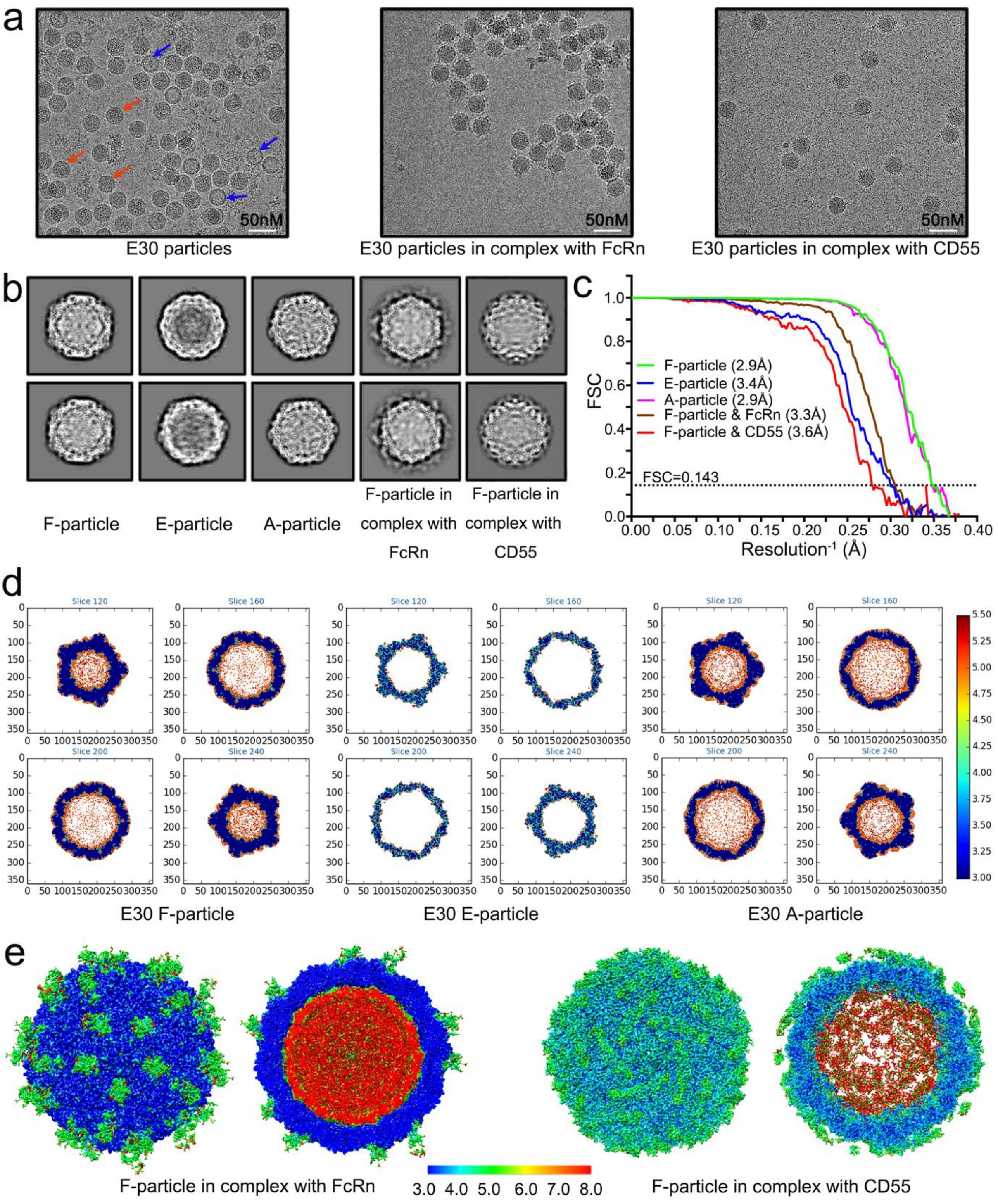

Supplementary Figure 3

Cryo-EM Images and Map Resolution Evaluation. **(a)** Cryo-EM micrographs of E30 particles (left) with F-particles and E-particles marked with red and blue arrows, respectively, F-particle in complex with FcRn (middle) and F-particle in complex with CD55 (right). **(b)** Representative classes from 2D classification in Relion 3.0[1]. **(c)** Gold-standard Fourier shell correlation (FSC) curves of the final maps of E30 F-particle, E-particle, A-particle, F-particle in complex with FcRn and F-particle in complex with CD55[2]. **(d)** Final map resolution

assessment of the slices through structures of the particles – E30 F-particle (left), E-particle (middle) and A-particle (right). **(e)** Final map resolution assessment of the structures of E30 F-particle in complex with FcRn (left) and E30 F-particle in complex with CD55 (right).

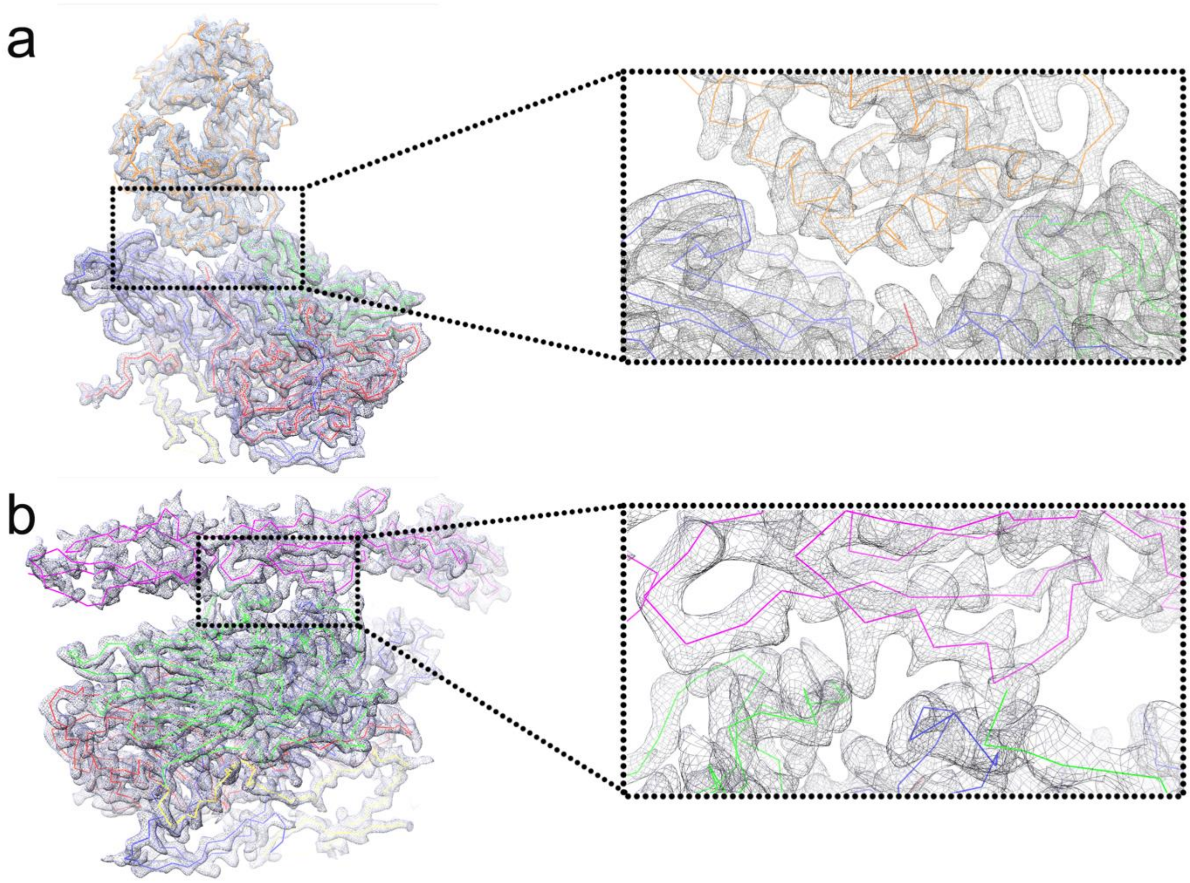

Supplementary Figure 4.
EM density maps for the virus-receptor-complexes. (a) EM density maps for the overall E30-FcRn-complex and the receptor binding interface between E30 and FcRn. (b) EM density maps for the overall E30-CD55-complex and the receptor binding interface between E30 and CD55.

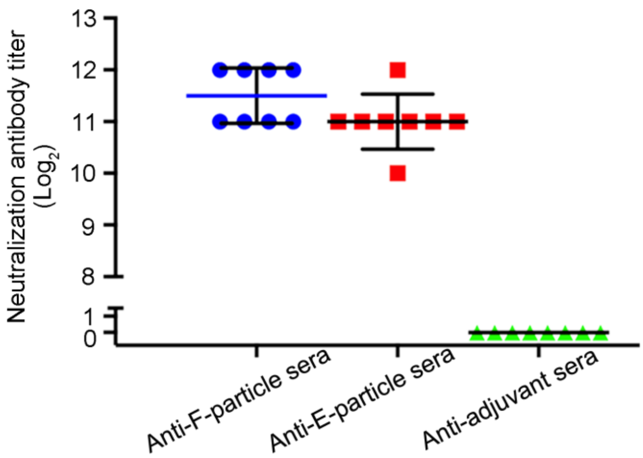

Supplementary Figure 5

Neutralization test of antisera against E30 F-particle and E-particle. Both sera obtained by immunizing mice with E30 F-particle and E-particle could neutralize E30 with high titration, whilst sera obtained from adjuvant-immunized mice failed to protect RD cells from E30 infection.

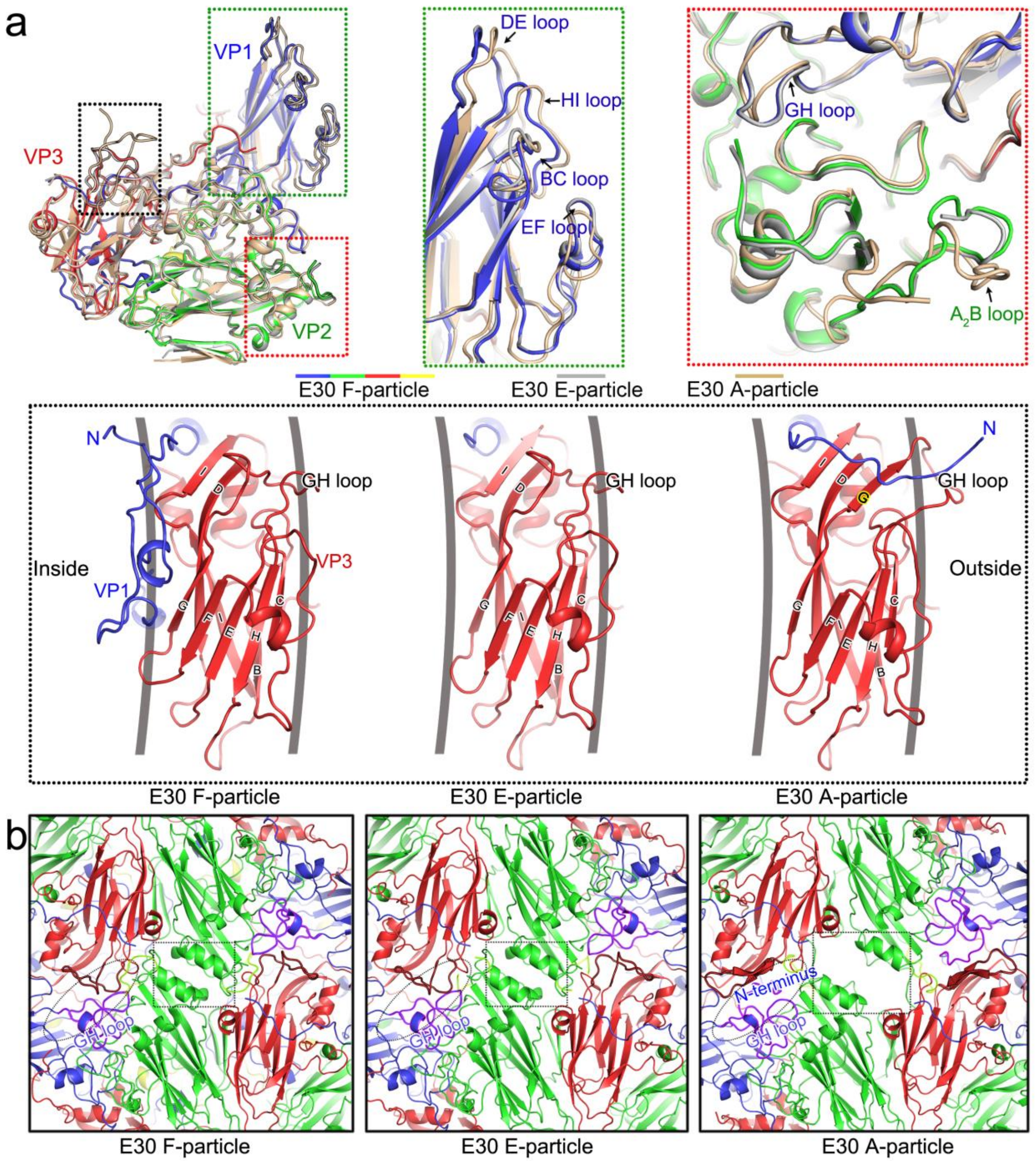

Supplementary Figure 6

Major Distinctions Between the E30 F-, E- and A-particles. (a) Superposition of the protomeric units from E30 F- (signature color scheme - VP1, blue; VP2, green; VP3, red), E- (grey) and A-particle (wheat); icosahedral symmetry axes are drawn in black. The parts exhibiting the most prominent alterations in VP1 and VP2 are highlighted in colored insets and zoomed in; the structural alterations in VP1 and VP3 out of the particle expansion and VP1 egress are highlighted in black inset. **(b)** Close-up views centered on the twofold axes of E30 F- (left), E- (middle) and A-particle (right). While the twofold channel in dotted rectangles on A-particle is open, the counterparts on F-particle and E-particle are closed.

Similar to the twofold channels, the off-axis channels in dotted ellipses are closed in F- and E- particle, and unobstructed in A-particle. VP1 N-terminus and VP1 GH loop, VP2 A_2B loop, VP3 GH loop are outlined in magenta, yellow and black, respectively.

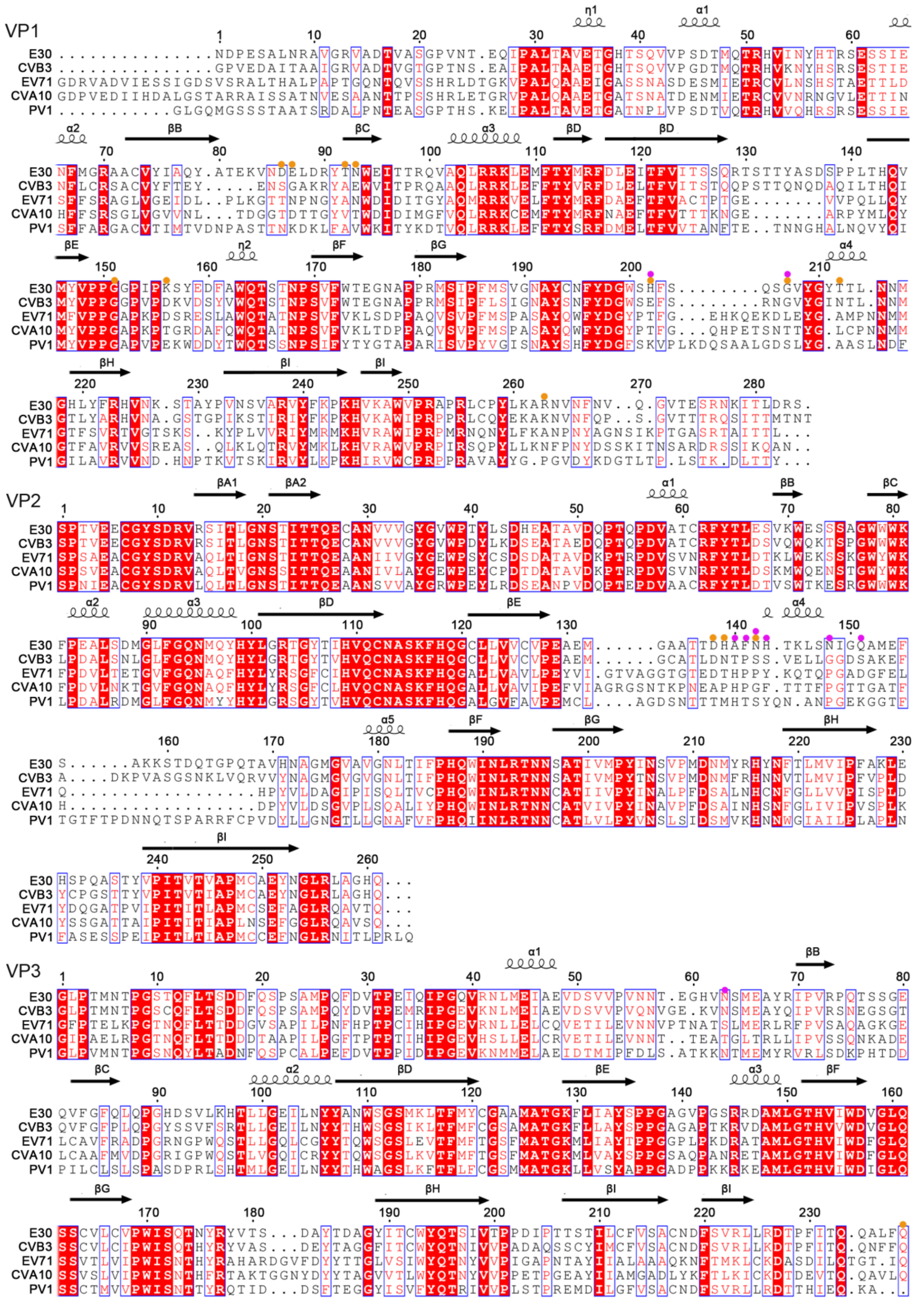
VP1
VP2
VP3
E30
CVB3
EV71
CVA10
PV1

Supplementary Figure 7

Espript[3] Representation of Sequence (of enterovirus A, B and C) Alignment. The sequence of E30 viral proteins were aligned with those from representative members of enterovirus A (EV71, CVA10), B (CVB3) and C (PV1). The residues involved in the interaction with uncoating receptor FcRn and attachment receptor CD55 are marked with balls in orange and magenta, respectively.

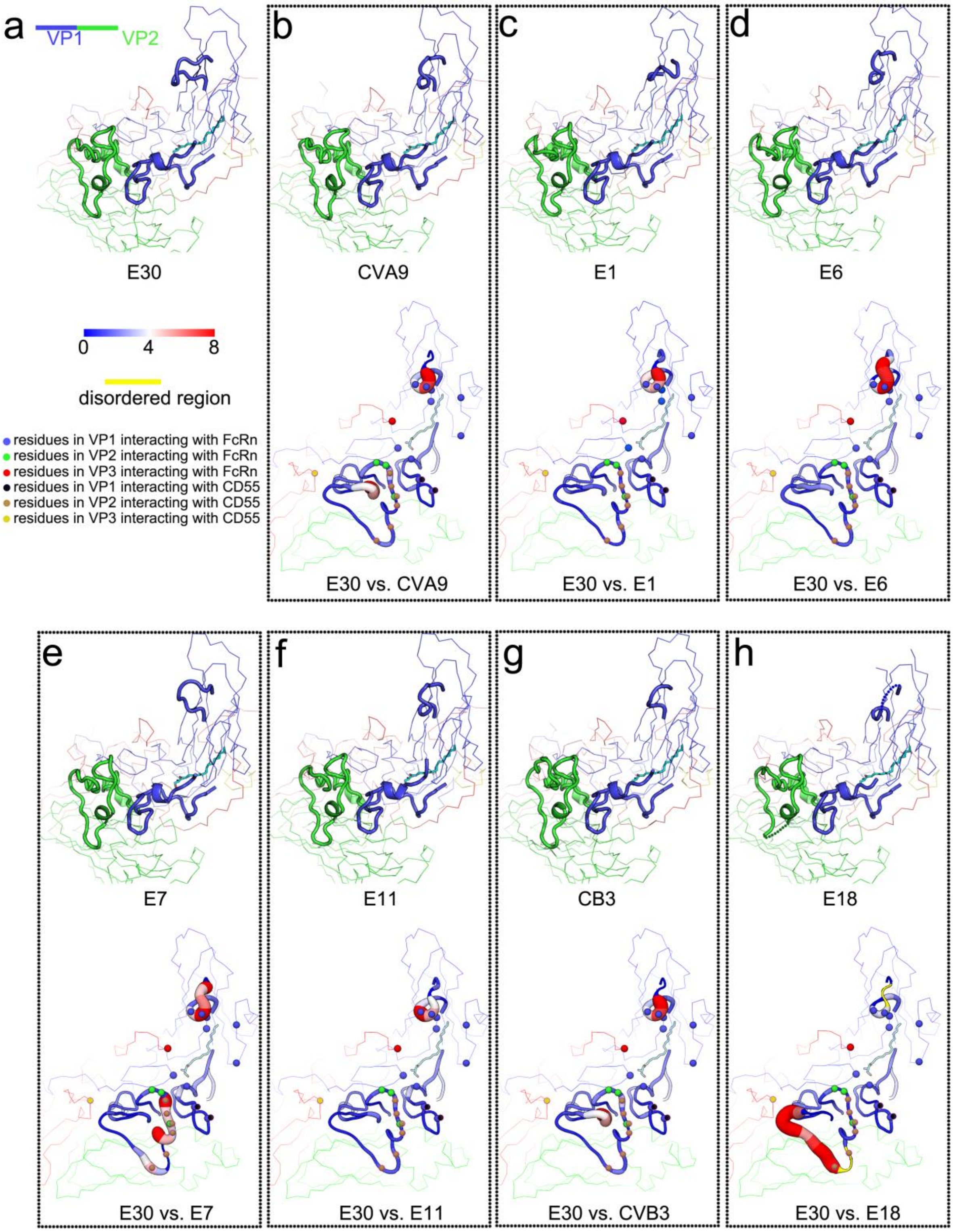

Supplementary Figure 8

Structure Comparisons of Enterovirus B. The protomeric units of each members in enterovirus B (E30; CVA9, 1D4M; E1, 1EV1; E6, 6ILP; E7, 2X5I; E11, 1H8T; CVB3, 1COV; E18, 8HBG) are exhibited with loops (VP1 BC loop, VP1 GH loop and VP2 EF loop) forming the canyon walls shown as cartoon and the rest as lines (upper part in each inset). Then the structures from above were superposed on E30 individually and the structural

differences were mapped onto the protomer of E30; the thickness and color of the worm representation reflect the local deviation between the structures (from blue (0 Å) to red (8 Å)) in the lower part in each inset. Also, residues in VP1, VP2 and VP3 that interact with FcRn are shown as blue, green and red spheres, respectively; and residues involved in the interaction with CD55 are shown as black, brown and yellow spheres, respectively.

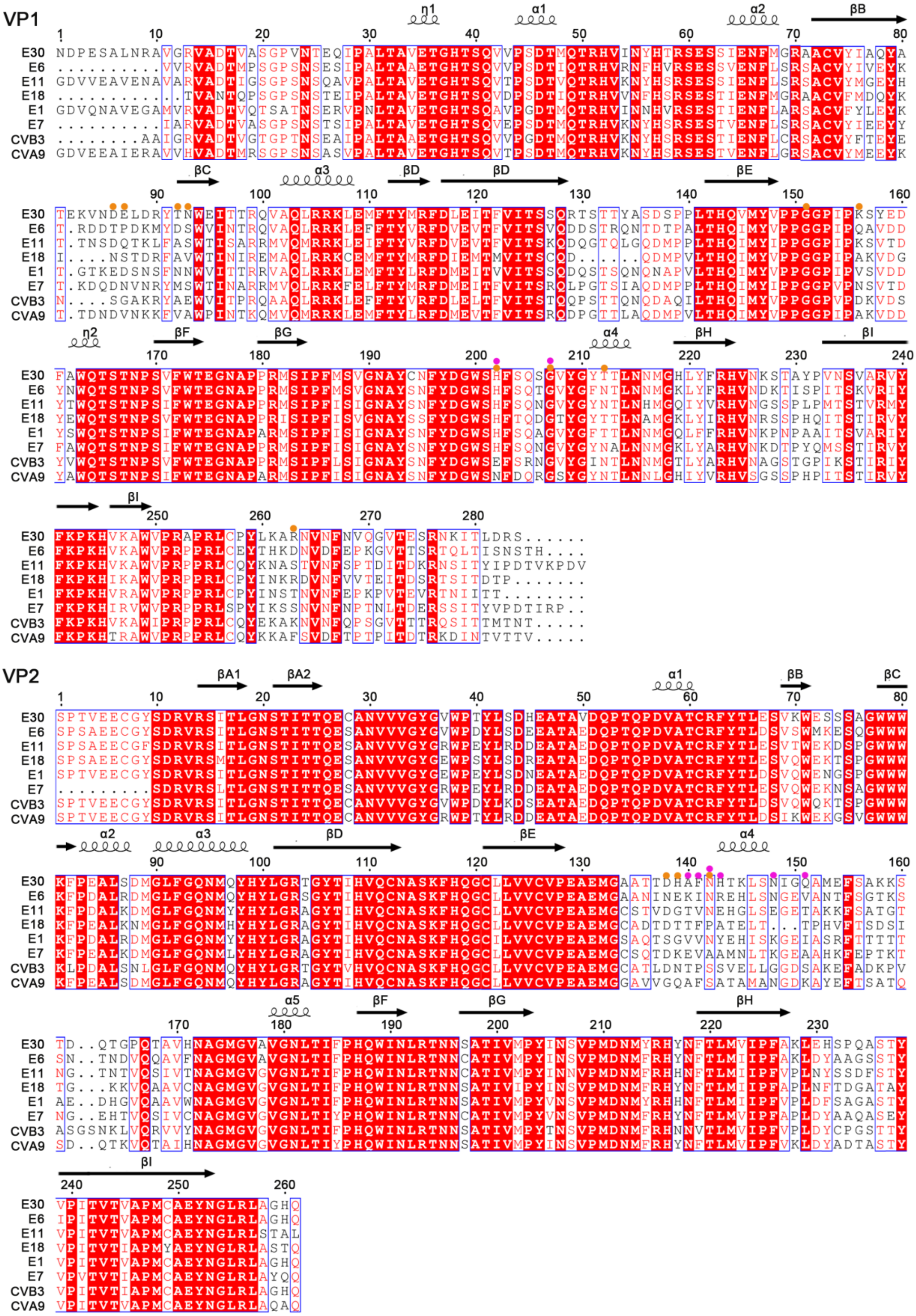
VP1
VP2

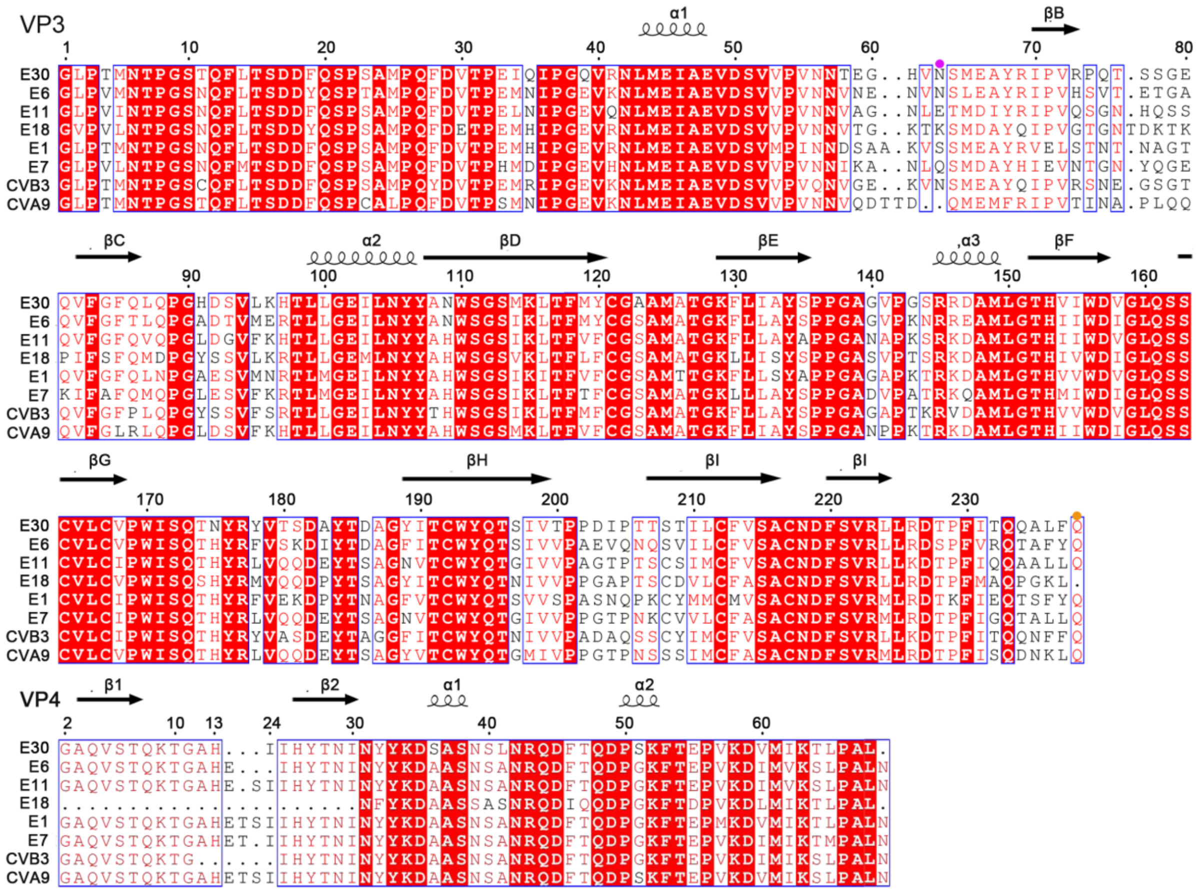

Supplementary Figure 9

Espript[3] Representation of Sequence (of enterovirus B) Alignment. The sequence of E30 viral proteins were aligned with other representative members (E3, E11, E16, E1, E7, CVB3 and CVA9) of enterovirus B in (b). The residues involved in the interaction with uncoating receptor FcRn and attachment receptor CD55 are marked with balls in orange and magenta, respectively.

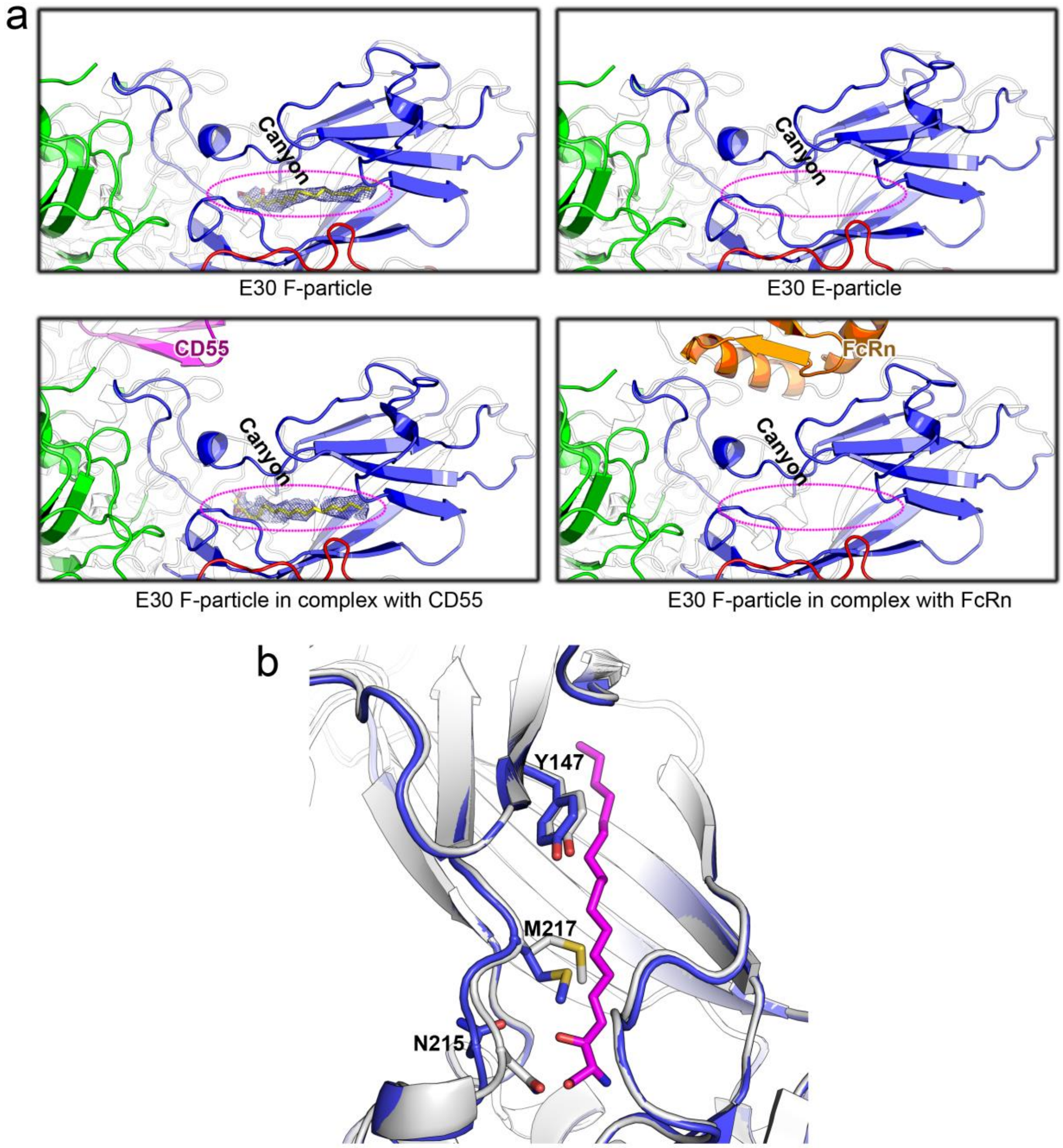

Supplementary Figure 10

Release of pocket factor upon the binding of FcRn. (a) Structures around the hydrophobic pocket of E30 F-particle, E30 E-particle, F-particle-CD55-complex and F-particle-FcRn-complex are compared. The viral proteins are shown as cartoon with the signature colors (VP1, blue, VP2, green, VP3, red), CD55 and FcRn are also shown as cartoon in magenta and orange, respectively. The hydrophobic pockets of the structures are circled in dotted lines in magenta and the pocket factors of E30 F-particle and E30 F-particle in complex with CD55 are shown as sticks with electron densities around them. In addition, no pocket factors are observed in E30 E-particle and E30 F-particle complexed with FcRn. **(b)** Superposition of

the pocket factor-binding regions of E30 F-particle (colored in blue) and F-particle-FcRn-complex (colored in grey); The pocket factor in F-particle are shown as sticks in magenta.

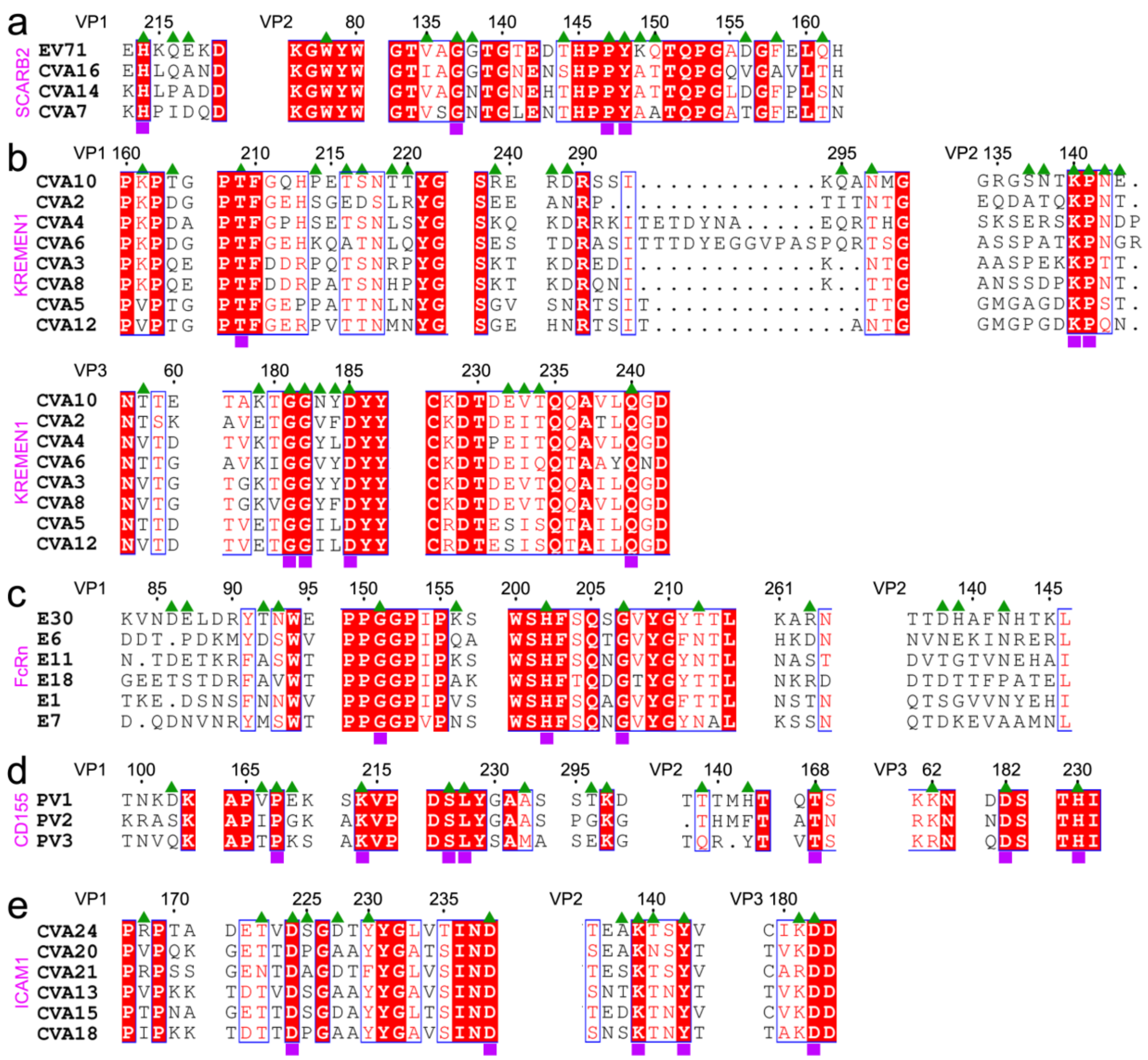

Supplementary Figure 11

Conservation analysis of viral receptor binding domains. Espript[3] representation of the sequence alignment of receptor binding domains for 5 subgroups with SCARB2 (a), KREMEN1 (b), FcRn (c), CD155 (d) and ICAM1 (e), respectively as their uncoating receptors. The residues that are recognized by their uncoating receptors are marked with triangles in green, among which the ones conserved across all strains are marked with boxes in purple.

Supplementary Table 1 **Statistics for Cryo-EM imaging, data processing and refinement of models.**

Name	F-particle	E-particle	A-particle	F-particle in complex with FcRn	F-particle in complex with CD55
**Data collection**					
Microscorpe	FEI Titan Krios				
Camera	Gatan K2				
Voltage (kV)	300				
Total dose (e^-/Å^2)	30				
Symmetry imposed	I	I	I	I	I
Micrographs (total)		1,404		1,084	537
Micrographs (used)		957		892	452
Particles selected		60,817		42,039	3,096
Particles included in final reconstruction	19,272	16,676	2,406	7,299	1,016
**Reconstruction**					
Sampling, Åper pixel	1.347	1.347	1.347	1.316	1.314
Defocus range (μm)	1.2 - 2.5	1.2 - 2.5	1.2 - 2.5	1.2 - 2.5	1.2 - 2.5
Resolution (Å) (FSC = 0.143 criterion)	2.9	3.4	2.9	3.3	3.6
**Model Refinement**					
Clashscore	3.61	3.92	4.13	7.02	4.47
Rotamer outliers (%)	0.14	0.47	0.32	0.78	0.45
Molprobity score	1.44	1.61	1.63	1.86	1.69
**Ramachandran statistics (%)**					
Most favored (%)	95.81	93.31	93.29	92.48	92.51
Allowed (%)	4.19	6.69	6.71	7.44	7.39
Outliers (%)	0	0	0	0.09	0.10
**R.m.s.deviations**					
Bond lengths (Å)	0.008	0.006	0.007	0.010	0.006
Bond angles (°)	0.811	0.741	0.856	0.902	0.849

Supplementary Table 2 **List of interactions between E30 and FcRn.**

E30			FcRn		
Location	Domain	Residues	Residues	Distance	Type of interaction
VP1	BC	T92 [OG1]	K146 [NZ]	4.00	hydrogen bond
		N93 [OD1]	N149 [ND2]	2.78	
	EF loop	G151 [N]	Q124 [OE1]	3.54	
		K156 [NZ]	L122 [O]	2.16	
		K156 [NZ]	K123 [O]	3.30	
	GH loop	H202 [NE2]	W131 [O]	3.08	
		G207 [N]	P132 [O]	3.41	
		T212 [OG1]	Q139 [NE2]	3.98	
	C-terminus	R263 [NH1]	Q139 [OE1]	3.53	
VP2	EF loop	D138 [O]	R140 [NH2]	2.61	
		D138 [O]	K80 [NZ]	3.32	
		D138 [OD2]	K80 [NZ]	2.40	
		H139 [NE2]	F79 [O]	2.29	
		N142 [ND2]	G83 [O]	3.34	
VP3	C-terminus	Q238 [NE2]	D145 [OD2]	1.65	
VP1	BC loop	D86	K150	2.86	electrostatic interaction
		E87	K150	3.82	
VP2	EF loop	D138	R140	2.61	
		D138	K80	2.40	
VP2	EF loop	D138 [OD2]	K80 [NZ]	2.40	salt bridge

Distances and types of interactions were first calculated by PISA program of software CCP4[4,5], and further manually verified and corrected by software Coot[6].

Supplementary Table 3 **List of interactions between E30 and CD55.**

E30			CD55		
Location	Domain	Residues	Residues	Distance	Type of interaction
VP1	GH loop	H202 [NE2]	V144 [O]	3.00	hydrogen bond
		G207 [N]	D143 [OD1]	2.94	
VP2	EF loop	F141 [O]	Q141 [NE2]	3.72	
		N142 [ND2]	C158 [O]	1.17	
		Q151 [OE1]	R214 [NH1]	1.54	
		Q151 [OE1]	R214 [NE]	2.62	
		Q151 [OE1]	R214 [NH2]	3.29	
		H143 [N]	Q141 [OE1]	2.50	
		N148 [ND2]	T160 [OG1]	3.49	
VP3	N-terminus	N63 [ND2]	P105 [O]	3.11	

Distances and types of interactions were first calculated by PISA program of software CCP4[4,5], and further manually verified and corrected by software Coot[6].

Supplementary References

1. Scheres, S. H. W. RELION: implementation of a Bayesian approach to cryo-EM structure determination. *J. Struct. Biol.* **180,** 519–530 (2012).
2. Scheres, S. H. W. & Chen, S. Prevention of overfitting in cryo-EM structure determination. *Nat. Methods* **9,** 853–854 (2012).
3. Robert, X. & Gouet, P. Deciphering key features in protein structures with the new ENDscript server. *Nucleic Acids Res.* **42,** W320–4 (2014).
4. Potterton, E., Briggs, P., Turkenburg, M. & Dodson, E. A graphical user interface to the CCP4 program suite. *Acta Crystallogr. D Biol. Crystallogr.* **59,** 1131–1137 (2003).
5. Winn, M. D. *et al.* Overview of the CCP4 suite and current developments. *Acta Crystallogr. D Biol. Crystallogr.* **67,** 235–242 (2011).
6. Emsley, P. & Cowtan, K. Coot: model-building tools for molecular graphics. *Acta Crystallogr. D Biol. Crystallogr.* **60,** 2126–2132 (2004).
